# Sensorimotor and Posterior Brain Activations During the Observation of Illusory Embodied Fake Hand Movement

**DOI:** 10.3389/fnhum.2019.00367

**Published:** 2019-10-15

**Authors:** Satoshi Shibuya, Satoshi Unenaka, Takuro Zama, Sotaro Shimada, Yukari Ohki

**Affiliations:** ^1^Department of Integrative Physiology, School of Medicine, Kyorin University, Tokyo, Japan; ^2^Department of Sport Education, School of Lifelong Sport, Hokusho University, Ebetsu, Japan; ^3^Department of Electronics and Bioinformatics, School of Science and Technology, Meiji University, Kawasaki, Japan

**Keywords:** body ownership, delayed visual feedback, independent component analysis, electroencephalography, mu-rhythm desynchronization, alpha-rhythm desynchronization, rubber hand illusion

## Abstract

In the rubber hand illusion (RHI), the subject recognizes a fake hand as his or her own. We recently found that the observation of embodied fake hand movement elicited mu-rhythm (8–13 Hz) desynchronization on electroencephalography (EEG), suggesting brain activation in the sensorimotor regions. However, it is known that mu-rhythm desynchronization during action observation is confounded with occipital alpha-rhythm desynchronization, which is modulated by attention. This study examined the independence of brain activities in the sensorimotor and occipital regions relating to the movement observation under the RHI. The invisible participant’s left and fake right hands were stroked simultaneously, which was interrupted by unexpected fake hand movements. A mirror-reversed image of the fake hand was shown on a monitor in front of the participant with a delay of 80, 280, or 480 ms. Illusion strength decreased as a function of the delay. EEG independent component analysis (ICA) and ICA clustering revealed six clusters with observation-induced desynchronization of 8–13 Hz frequency band. In the right sensorimotor cluster, mu-rhythm desynchronization was the greatest under the 80-ms delay, while alpha-rhythm desynchronization of the occipital clusters did not show delay-dependence. These results suggest that brain activation in the sensorimotor areas (i.e., mu-rhythm desynchronization) induced by embodied fake hand movement is independent of that in the occipital areas (alpha-rhythm desynchronization).

## Introduction

Sense of body ownership describes the subjective feeling that one’s body belongs to oneself and is a fundamental aspect of one’s sense of self (Gallagher, 2000, 2005). Although body ownership is generally rigid, accumulating findings from research implementing the RHI suggest that body ownership is flexible even in healthy individuals and can be projected onto a non-corporal object. In the traditional visuo-tactile RHI (Botvinick and Cohen, 1998), an experimenter repeatedly strokes a visible fake hand (i.e., rubber hand) and an invisible participant’s hand in synchrony, causing the participant to experience illusory ownership of the fake hand. The experience of RHI is generally assessed using a questionnaire and by observing a phenomenon known as proprioceptive drift in which the subject’s proprioceptive perception shifts toward the fake hand under the illusion. If two hands are stroked in asynchrony, the RHI is reduced or abolished (Ehrsson et al., 2004; Tsakiris et al., 2007), suggesting that temporal consistency between visual and tactile stimuli is necessary to induce the RHI.

During the RHI, it is known that action observations of the embodied fake hand inversely affect the sensorimotor system controlling the participant’s hand (Schutz-Bosbach et al., 2006; Slater et al., 2008). For example, Slater et al. (2008) performed synchronous or asynchronous stroking of the real and virtual (fake) hands, which was terminated after 5 min of stimulation and followed by fake hand movement. When participants experienced illusory ownership of the fake hand in the synchronous condition, action observation could induce activities in a muscle relevant to the observed action, which were less observed in the asynchronous condition. Our group recently used EEG to demonstrate the inverse effects on sensorimotor areas by action observation in the embodied fake hand (Shibuya et al., 2018). For that purpose, mu-rhythm (8–13 Hz) desynchronization was observed, which is induced during movement execution and action observation (Oberman et al., 2005; Pineda, 2005). Similar to the previous works, we found that participants often exhibited spontaneous hand movements in accordance with the fake hand movement during the RHI. Additionally, the action observation induced larger and more persistent mu-rhythm desynchronization in the sensorimotor areas under RHI, suggesting a relationship between mu-rhythm desynchronization and the RHI.

Nevertheless, it is possible that differences in mu-rhythm desynchronization in our previous study were related to higher attentional allocation to the embodied compared to non-embodied fake hand movement. In fact, a recent neurological study by Aizu et al. (2018) demonstrated that the body-specific visual attention to the paretic hand was lower in stroke patients than healthy subjects due to learned non-use, which might be linked to reduced ownership of the paretic hand (Burin et al., 2015, 2017). In a related EEG study, Perry and Bentin (2010) suggested that desynchronization at ∼10 Hz induced by action observation depended on attentional demand because EEG power reduction at central (C3 and C4 of the international 10–20 system; mu-rhythm) and occipital electrodes (O1 and O2; alpha-rhythm) showed similar patterns and were modulated by task difficulty. Given the volume conduction, mu-rhythm activity recorded from the sensorimotor region may be contaminated by posterior alpha-rhythm activity (Hobson and Bishop, 2016, 2017).

The current study investigated the dependence/independence of brain activities in the sensorimotor areas (i.e., mu-rhythm desynchronization) related to the movement observation under the RHI and those in the occipital areas (alpha-rhythm desynchronization) reflecting attention. For that purpose, we used a similar setup to that in our previous study (Shibuya et al., 2018), and applied an ICA and ICA clustering to the resultant EEG data (Makeig et al., 2002; Delorme and Makeig, 2004), which allowed us to judge the independence and dependence of signals. Additionally, we introduced three visual feedback delays during the illusion induction (i.e., 80, 280, or 480 ms), which might induce different effects on attention and the RHI, compared to the synchronous/asynchronous stimulation in our previous study. We predicted that the sensorimotor mu-rhythm and the occipital alpha-rhythm component were modulated differently depending on the feedback delay.

## Materials and Methods

### Participants

The study included 33 healthy participants (eight men and 25 women; mean age ± standard deviation, 22.1 ± 6.3 years). Participants were blinded to the purpose of the experiment and all but four were right-handed as per the Edinburgh Inventory (Oldfield, 1971).

### Experimental Apparatus

**FIGURE 1:**
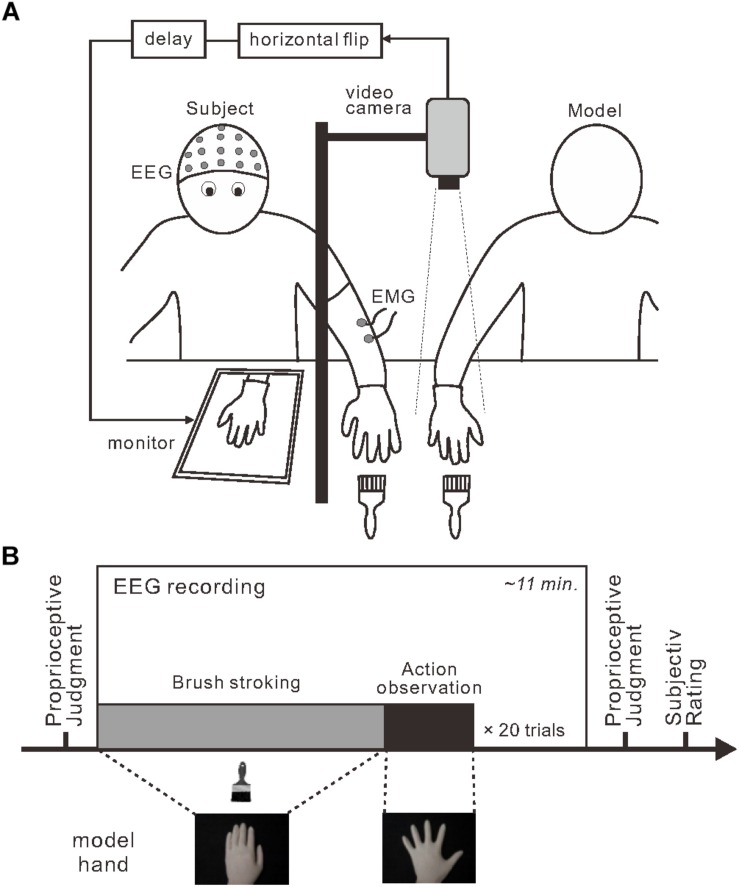
Experimental setup and design. **(A)** To induce the rubber hand illusion, an experimenter stroked a participant’s visually occluded left hand and a model’s right hand simultaneously. Participants viewed a monitor that displayed the horizontally flipped model hand. Three delay intervals were introduced during stroking: 80, 280, and 480 ms. **(B)** Each session (box) included a stroking period (gray) followed by an action-observation period (black) that were repeated. Before and after each session, the participants completed a proprioceptive judgment about their own left hand to assess proprioceptive drift.

Participants wore a latex glove on their left hand and placed the hand on a desk (Figure 1A). A 15-inch tablet monitor (On-lap 1503H, Gechic Corp., Taichung, Taiwan) was positioned on the desk facing upward in front of the participant. A partition (black board) placed between the participant’s hand and the monitor prevented direct view of the participant’s hand. An experimenter’s right hand (model hand), also wearing a latex glove, was positioned next to the participant’s left hand. Using a video camera with a rate of 30 frames per second, top-view video of the model hand was captured, flipped horizontally, delayed (only in the 280 and 480-ms delay conditions) and displayed on the monitor. The lateral distance between participant’s hand and model’s hand image was maintained at 25 cm across the experiment. No artificial visual feedback delay was introduced in the 80-ms delay condition; the inherent delay of approximately 80 ms was below the threshold for detecting visual feedback delay (Shimada et al., 2010). In contrast, visual feedback delays of 200 and 400 ms were added in the 280 and 480-ms conditions to produce actual perceptible time delays of 280 and 480 ms, respectively.

### Procedure

Each participant completed six experimental sessions in which they encountered each delay condition twice. The order of conditions was randomized in the first three sessions and the order was repeated for the last three sessions. Each session consisted of 20 combinations (trials) of the stroking phase and the model hand movement phase (i.e., action observation) (Figure 1B). Before the first session, participants were instructed to watch the model hand and to remain as relaxed as possible throughout the session. Participants were not informed about unexpected movements of the model hand. During stroking phase, the experimenter stroked both the model hand and the participant’s hand simultaneously at 0.5 Hz using paintbrushes. Assuming a typical duration to RHI onset after stroking (11 ± 7 s; Ehrsson et al., 2004), the duration of the stroking phase was pseudo-randomly assigned to be 16, 20, 24, 28, or 32 s. During the model hand movement phase, the experimenter terminated the stroking movements and the model hand performed a single abduction and adduction movement with the fingers of the whole hand. The movement duration and time to maximally open the hand were approximately 6 and 3 s, respectively. We detected the initiation of model hand movement using a mechanical touch switch (STM6, Asa Electronics Industry Co., Tokyo, Japan) and this signal was used as a trigger for the EEG analysis.

Participants provided a proprioceptive judgment of their left-hand position before and after each session to estimate proprioceptive drift. Participants were instructed to close their eyes and the experimenter removed the partition and placed a board (60 cm × 45 cm) with a 6-cm ruler over the participant’s hand and the monitor. Subsequently, participants were asked to open their eyes and verbally report the perceived position of the visually occluded left middle fingertip. To avoid repetition of response values from previous trials, the experimenter offset the ruler in each trial. Proprioceptive drift was computed by subtracting the pre-test value from the post-test value for each session and then averaged across two sessions with the same condition.

After the second proprioceptive judgment in each session, participants were asked to report their subjective feelings during the session using a questionnaire. The questionnaire was identical to that used in our previous study (Shibuya et al., 2018) and consisted of nine items (Table 1): Q1–Q8 were items based on previous studies (Botvinick and Cohen, 1998; Kalckert and Ehrsson, 2012) to check illusory body ownership and agency over the model hand (and model hand movement), while Q9 checked whether the participants were aware of movement of their own hand during observation of the model hand movement. These items were divided into five categories; *Ownership* (Q1 and Q2), *Ownership control* (Q3 and Q4), *Agency* (Q5 and Q6), *Agency control* (Q7 and Q8), and *Awareness* (Q9). Participants responded to each item using a 7-point Likert scale that ranged from +3 (strongly agree) to -3 (strongly disagree). After completing the questionnaire, participants were allowed a 5-min rest period before starting the next session. As for categories other than *Awareness*, we calculated the ratings by averaging those from two questions.

**TABLE 1:** Questionnaire.

**Category**	**Question**
Ownership	(1) It seemed as though the touch I felt was caused by the paintbrush touching the model hand (2) I felt as if the model hand were my hand
Ownership control	(3) It seemed as if I might have more than one left hand or arm (4) It felt as if I no longer had a left hand, as if my right hand had disappeared
Agency	(5) I felt as if I was controlling the movements of the model hand (6) I felt as if I was causing the movements of the model hand
Agency control	(7) I felt as if the model hand was controlling my hand movements. (8) I felt as if the model hand was controlling my will
Awareness	(9) I felt as if my hand was moving against my will during the observation of model hand movement

### Finger Movement Analysis

A commercial video camera (CX-485, Sony, Tokyo, Japan) was used to capture video (60 frames per second) of both the model hand and the participant’s hand during each session. One experimenter watched the videos and counted the number of trials in which participants’ fingers exhibited obvious movement within 6 s of the onset of model hand movement.

### EEG Data Acquisition and Analysis

Electroencephalography data during action observation was acquired at 512 Hz using a 32-channel EEG signal amplifier (Eego Sports, ANT-Neuro, Enschede, Netherlands) in accordance with the international 10–20 system. Scalp impedance was maintained below 15 kΩ and checked before and after each session. EEG signals were referenced to CPz with AFz grounds during data acquisition and re-referenced offline to the average of the left and right mastoids. Trigger onset was the time of movement initiation of the model hand image. EEGLAB (version 13.5.4b) was used to analyze all EEG data (Delorme and Makeig, 2004). Recorded EEG data were preprocessed in accordance with predetermined steps: first, raw data were downsampled to 256 Hz and subjected to a 1–40-Hz band-pass filter. Second, data were segmented into epochs from 1000 ms pre-onset to 5000 ms post-onset. Third, bad epochs were automatically discarded in accordance with predetermined criteria: (1) the epoch included an amplitude exceeding ±200 μV; or (2) the epoch included a power spectrum exceeding 3 standard deviations from the mean.

We next excluded epochs containing phasic muscle activity during observation of the model hand movement using surface electromyogram (EMG) measured from the extensor digitorum communis (EDC) and extensor pollicis longus (EPL) of the participant’s left arm using bipolar recording. The raw EMG signal was amplified, filtered using a 50-Hz notch filter and 10-Hz high-pass filter, digitized at 512 Hz, and rectified. We automatically discarded epochs in which EMG amplitudes for the EDC and/or EPL within 3 s from movement onset exceeded 3 standard deviations of the mean during 1000 ms pre-onset. Next, we visually inspected EMG profiles for each epoch to remove epochs wherein obvious phasic responses were observed within 5 s from the onset. Consequently, 7.5 ± 5.4 epochs (18.7 ± 13.4%), 7.2 ± 4.6 epochs (18.1 ± 11.4%), and 5.9 ± 3.8 epochs (14.8 ± 9.4%) of all 40 epochs were excluded in the 80, 280, and 480-ms delay conditions, respectively. No significant effect of delay intervals on excluded epochs was detected (*p* > 0.1; one-way ANOVA).

After epoch rejection, an ICA was applied to preprocessed EEG data using the infomax ICA algorithm (Bell and Sejnowski, 1995), implemented in EEGLAB runica function and 30 components were obtained for each participant (total of 990 components across all participants). Subsequently, the locations of equivalent current dipoles were estimated using the DIPFIT function (version 2.3) implemented in EEGLAB. To clean data, we excluded components with dipole residual variances >15% (i.e., low accuracy of the estimated dipole location) (Artoni et al., 2014), resulting in the use of 523 components (53%) of 990 for further ICA clustering.

For group data analysis, ICA clustering was performed across subjects using the *k*-means algorithm (MacQueen, 1967) in accordance with the spatial topographic map, dipole location, and ERSP as determination factors in EEGLAB STUDY function. The *k*-means is one of the simplest unsupervised learning algorithms for solving the clustering problem. With regard to generated clusters, we inspected the estimated centroid of dipole locations, the power spectrum, the total number of participants contributing to each cluster, and the number of components contributed by each participant to clusters. If a participant included multiple components in a single cluster, we selected the component with the lowest residual variance (Denis et al., 2017). Out of these clusters, the target cluster was selected using the following criteria: (1) more than 75% of participants (*n* ≥ 25) contributed to a cluster and (2) the power spectrum showed a prominent peak at about 10 Hz. We finally identified the Brodmann area (BA) of the dipole centroid location (BA within 4 mm of the most significant voxel) for each target cluster using Talairach Client (version 2.4.3) (Lancaster et al., 1997, 2000).

For the time-frequency analysis of each target cluster, we compared ERSPs of each target cluster in the 3–40 Hz frequency range between experimental conditions. First, we calculated the ERSP of each cluster during model hand movement compared to baseline (−0.5 to 0 s from onset) using a Morlet wavelet transform, implemented in EEGLAB. The wavelet increased from 3 cycles at the 3 Hz to 20 cycles at 40 Hz. Two hundred time points from −443 to 4439 ms and 38 linear-spaced frequencies from 3 to 40 Hz were generated. The window size was 1113 ms and a time resolution was 24 ms. Second, bootstrapped significance tests were used to evaluate significant differences in ERSP time-frequency data between conditions at the *p* < 0.01 level and corrected for multiple comparisons using false discovery rate control. Finally, we calculated the mean power in the 8–13 Hz frequency range from the onset of model hand movement to 4 s post-onset for each condition.

## Results

### Questionnaire Ratings and Proprioceptive Drift

**FIGURE 2:**
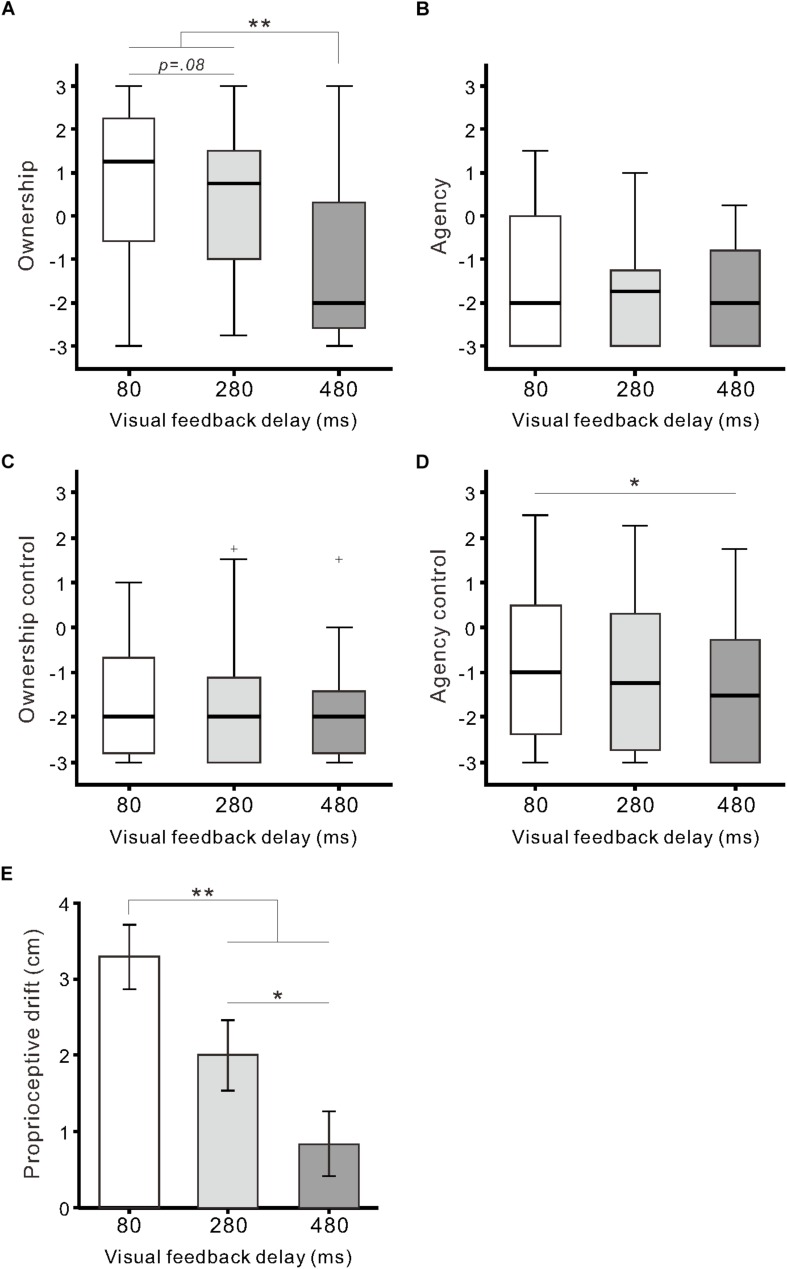
Questionnaire ratings and proprioceptive drift. Boxplots of questionnaire ratings against the delay conditions are shown for *Ownership* **(A)**, *Agency* **(B)**, *Ownership control* **(C)**, and *Agency control* **(D)**. Boxes and thick lines denote the interquartile ranges (IQRs) and medians, respectively. Whiskers represent either additional data points or extend to 1.5 × IQR. Small plus signs indicate outliers. **(E)** Mean proprioceptive drift against the delay conditions. Vertical lines denote ± 1.0 SE. Significance is denoted using asterisks (^∗∗^*p* < 0.01; ^∗^*p* < 0.05).

Regarding questionnaire ratings of body ownership and agency, the Friedman test demonstrated that the null hypothesis of equal medians across the three delay conditions was rejected in two out of four categories: *Ownership* (χ^2^_2_ = 27.0, *r* = 0.39, *p* < 0.01) and *Agency control* (χ^2^_2_ = 11.2, *r* = 0.14, *p* < 0.01). For *Ownership* (Figure 2A), the medians of the 80-ms (1.3) and 280-ms delay conditions (0.8) were significantly higher than that of the 480-ms delay condition (−2.0) (both, *p* < 0.01; Scheffe’s test). Additionally, there was a marginally significant difference between the 80 and 280-ms delay conditions (*p* = 0.08). For *Agency control* (Figure 2D), the median of the 480-ms delay condition was significantly lower than that of the 80-ms delay condition (*p* < 0.05), though all medians were less than 0. For *Agency* (Figure 2B) and *Ownership control* (Figure 2C), the null hypothesis was not rejected (both, *p* > 0.1).

Proprioceptive drift was significantly influenced by visual feedback delay (*F*_(__2__,__64__)_ = 17.7, η^2^ = 0.36, *p* < 0.01; one-way ANOVA; Figure 2E). *Post hoc* tests indicated that drift in the 80-ms delay condition (3.3 ± 2.4 cm) was significantly larger than that in the 280-ms (2.0 ± 2.6 cm) and 480-ms delay conditions (0.8 ± 2.4 cm) (both *p* < 0.01; Tukey’s honest significant difference [HSD] test). Additionally, there was a statistically significant difference between the 280 and 480-ms delay conditions (*p* < 0.05). Proprioceptive drift was moderately correlated with *Ownership* rating (*r* = 0.33, *p* < 0.01; *n* = 99; Pearson’s correlation coefficient) across the three conditions.

### Finger Movements During the Observation of Model Hand Movement

**FIGURE 3:**
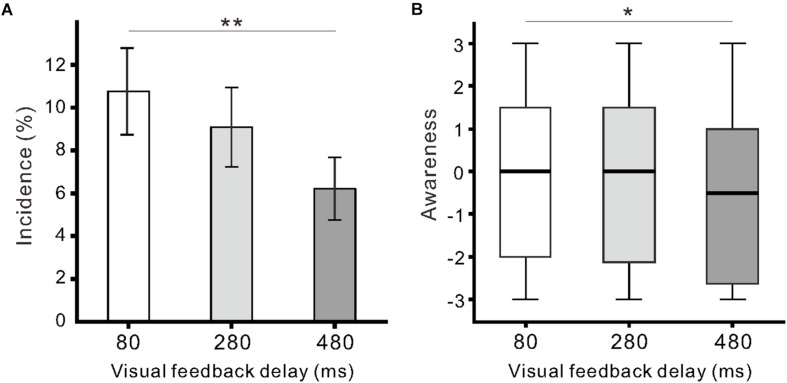
Participant finger movement evoked by the observation of model hand movement. **(A)** Mean incidence of finger movement against the delay conditions. Vertical lines denote ± 1.0 SE. **(B)** Boxplots of the questionnaire rating (*Awareness*) regarding finger movements. Significance is denoted using asterisks (^∗∗^*p* < 0.01; ^∗^*p* < 0.05).

Similar to our previous report (Shibuya et al., 2018), we found participants exhibited finger movement during the observation of model hand movement. Finger movements were observed in 29 of 33 participants across conditions, and the proportion of participants exhibiting movements (88%; 29/33) was larger than the proportion of participants not exhibiting movements (12%; 4/33; χ^2^_1_ = 18.9, *p* < 0.01; Chi-squared test). The incidence of finger movement was significantly influenced by visual feedback delay (*F*_(__2__,__64__)_ = 4.8, η^2^ = 0.13, *p* < 0.05; one-way ANOVA; Figure 3A). The incidence in the 80-ms delay condition (10.8 ± 11.6%) was significantly higher than that in the 480-ms delay condition (6.2 ± 8.4%; *p* < 0.01; Tukey’s HSD test). The incidence of finger movement was positively correlated with *Ownership* rating (*r* = 0.43, *p* < 0.01) and proprioceptive drift (*r* = 0.24, *p* < 0.05) across the three conditions. Subjective ratings of finger movements (*Awareness*) were not larger than 0 across all delay conditions (*p* > 0.08; one-sample *t*-test), though the null hypothesis of equal medians across the conditions was rejected (χ^2^_2_ = 7.6, *r* = 0.09, *p* < 0.05; Friedman test). There was a statistically significant difference between the 80 and 480-ms delay conditions (*p* < 0.05; Scheffe’s test; Figure 3B). In addition, the *Awareness* rating was positively correlated with the incidence of finger movement (*r* = 0.37, *p* < 0.01) and *Agency control* rating (*r* = 0.83, *p* < 0.01).

### EEG Data

#### ICA Clustering

**FIGURE 4:**
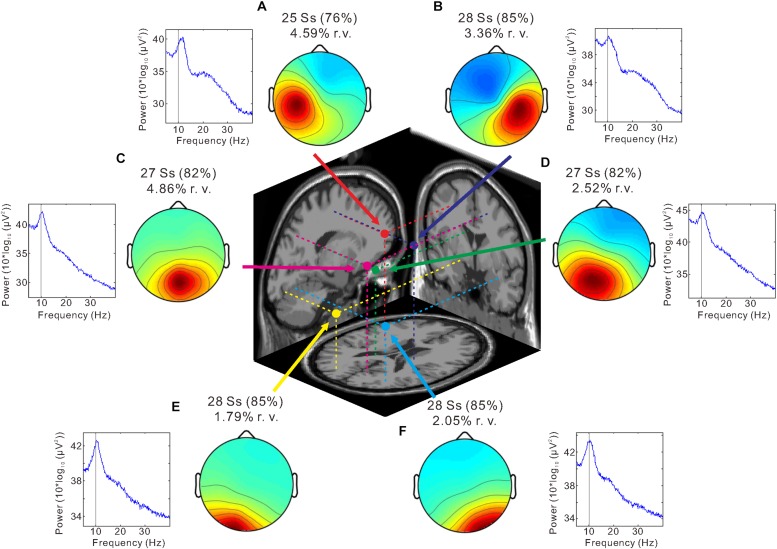
Independent component analysis (ICA) clustering. Scalp topography maps and estimated dipole centroid locations of the left sensorimotor cluster (**A**, red), right sensorimotor cluster (**B**, blue), parietal cluster (**C**, magenta), posterior cingulate cluster (**D**, green), left occipital cluster (**E**, yellow), and right occipital cluster (**F**, light blue) within a 3-shell boundary element model (BEM) of the Montreal Neurological Institute (MNI) standard brain. Graphs next to scalp maps display power spectra. Note that a peak is present at ∼10 Hz (thin vertical line) in all clusters. The number of subjects (Ss) contributing to each cluster and the mean residual variance (r. v.) are displayed above each scalp map.

We identified six clusters according to the criteria of the target cluster selection, which was described in Methods (Figure 4). Bilateral sensorimotor clusters were identified and their dipole centroid locations were localized to the left [Montreal Neurological Institute (MNI) coordinate: *x* = −34, *y* = 1, *z* = 42; BA 6] (Figure 4A, red) and right sensorimotor areas (MNI coordinate: *x* = 20, *y* = −15, *z* = 52; BA 6) (Figure 4B, blue) as well as the left (MNI coordinate: *x* = −20, *y* = −79, *z* = −6; BA 18) (Figure 4E, yellow) and right occipital clusters (MNI coordinate: *x* = 33, *y* = −66, *z* = −14; BA 19) (Figure 4F, light blue). The analysis also identified the parietal cluster (MNI coordinate: *x* = 7, *y* = −65, *z* = 48; right BA 7) (Figure 4C, magenta) and the posterior cingulate cluster (MNI coordinate: *x* = −9, *y* = −37, *z* = 24; left BA 23) (Figure 4D, green).

#### Differences Between Conditions in the Clusters

**FIGURE 5:**
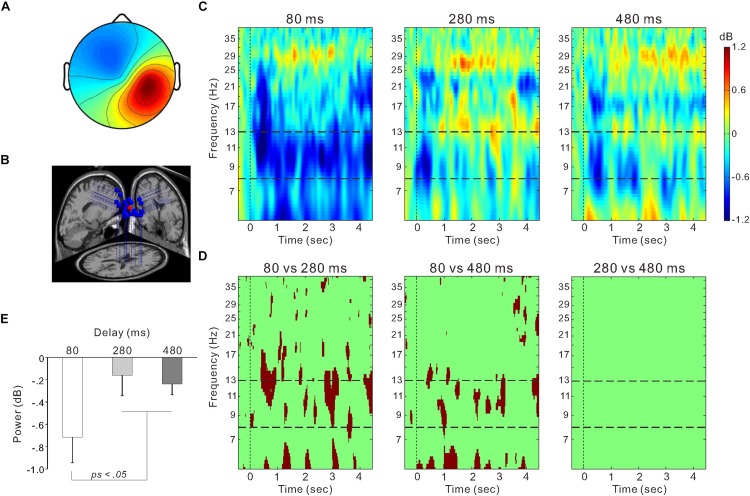
The right sensorimotor cluster. **(A)** Scalp topographical map. **(B)** Estimated dipole locations for each participant (blue) and their centroid locations (red). **(C)** Event-related spectrum perturbation (ERSP) time-frequency plots for the 80-ms (left), 280-ms (middle), and 480-ms delay conditions (right). Blue color indicates decreased power and red color indicates increased power. The band between two horizontal dashed lines indicates the mu-rhythm frequency range (i.e., 8–13 Hz). **(D)** Statistically significant differences in time-frequency plots (*p* < 0.01) between the 80 and 280-ms delay conditions (left), between the 80 and 480-ms delay conditions (middle), and between the 280 and 480-ms delay conditions (right) using the bootstrap method and false discovery rate correction. **(E)** Mean power within the 8–13 Hz frequency range from the onset of model hand movement to 4 s post-onset in each delay condition. Vertical lines denote standard error of the mean (SEM).

**FIGURE 6:**
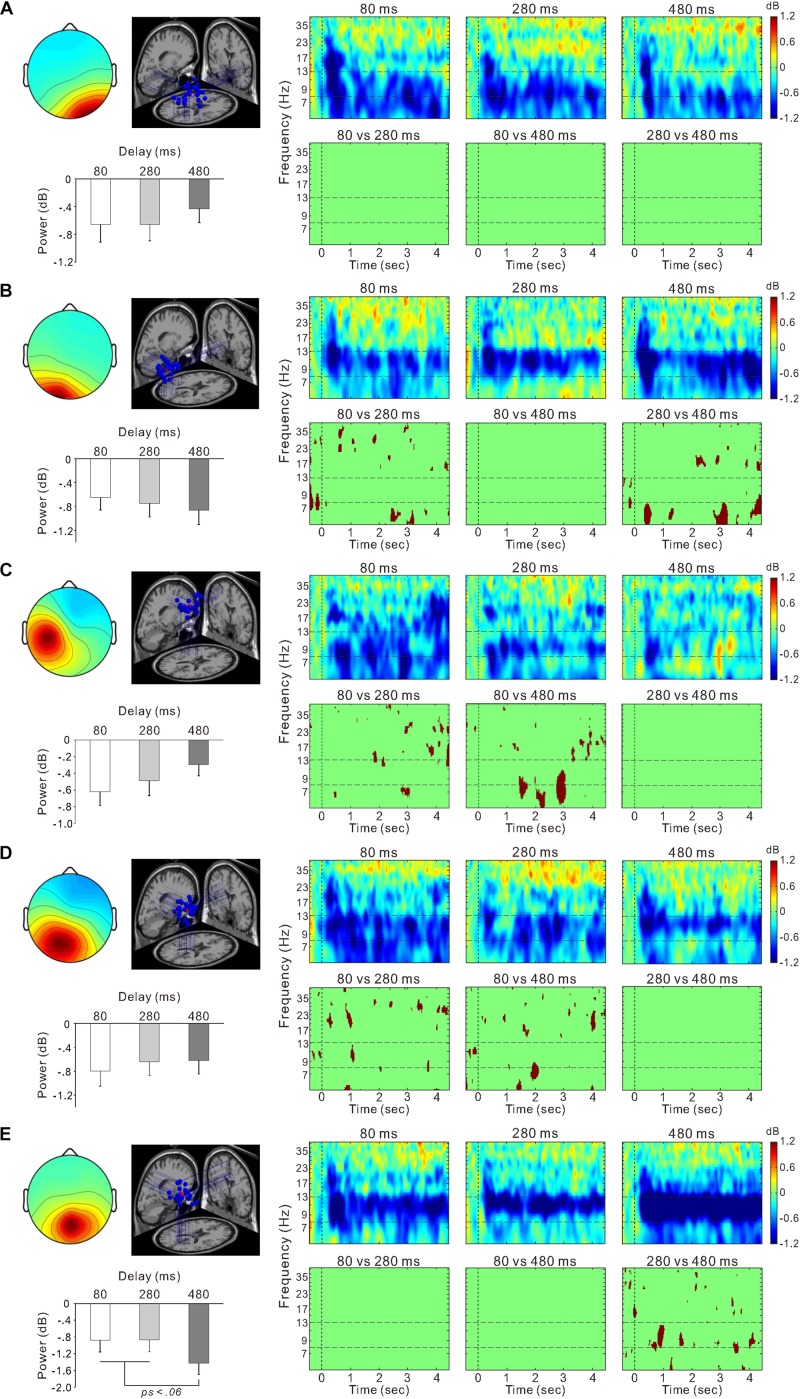
Scalp topographical map, dipole locations, ERSP time-frequency plot, and statistically significant differences in time-frequency plots and mean power within the 8–13 Hz frequency range for the right occipital cluster **(A)**, left occipital cluster **(B)**, left sensorimotor cluster **(C)**, posterior cingulate cluster **(D)**, and parietal cluster **(E)**.

We hypothesized that ERSP could be obtained in the right sensorimotor area, contralateral to the stroked hand, and the occipital area, which were independent from each other. Consistent with the hypothesis, we obtained an independent component cluster in the right sensorimotor cortex (Figures 4B, 5). We also observed clusters in the occipital cortex (Figures 4E,F, 6A,B), but there were two clusters in the left and right hemisphere, respectively. Next, we tested the second hypothesis that if the desynchronization within the 8–13 Hz frequency range (i.e., mu- and alpha-rhythm) declines as the visual feedback delay increased only in the right sensorimotor area.

Figure 5C shows ERSP time-frequency plots of the right sensorimotor cluster. Powers of the mu-rhythm (the band between the horizontal dashed lines) were decreased after the movement observation. Figure 5D presents amplitudes of the desynchronization between different delays, and the mean power of the mu-rhythm during 4 s from the onset of model hand movement was significantly influenced by visual feedback delay (one-way ANOVA; *F*_(__2__,__54__)_ = 4.8, η^2^ = 0.15, *p* < 0.05; Figure 5E). The desynchronization was significantly higher in the 80-ms delay condition (−0.72 ± 1.2 dB) than in the 280-ms (−0.16 ± 0.97 dB) and 480-ms delay conditions (−0.24 ± 0.52 dB; both *p* < 0.05; Tukey’s HSD test). There was no significant difference between the 280 and 480-ms delay conditions (*p* > 0.9). With regard to the occipital oscillations, we performed similar analyses for the two clusters (Figures 6A,B). Clear desynchronization was observed in both clusters, but there were no significant differences between conditions in the right (one-way ANOVA; *p* > 0.5; Figure 6A) and the left (*p* > 0.6; Figure 6B) occipital clusters. For the right sensorimotor cluster, we also performed correlation analyses between mu-rhythm desynchronization and ownership ratings or proprioceptive drift across conditions (*n* = 84); however, both results were non-significant (*p* > 0.7).

In the other clusters, we also observed the event-related desynchronization (Figures 6C–E). Thus, we further examined its modulation by the visual feedback delay. There were no significant differences between conditions in the left sensorimotor cluster (*p* > 0.2; one-way ANOVA; Figure 6C) and the posterior cingulate cluster (one-way ANOVA; *p* > 0.7; Figure 6D). However, the alpha-rhythm desynchronization of the parietal cluster (Figure 6E) tended to be affected by visual feedback delay (one-way ANOVA; *F*_(__2__,__52__)_ = 2.7, η^2^ = 0.09, *p* = 0.07). In contrast to the right sensorimotor cluster, desynchronization of the 480-ms delay (−1.4 ± 1.4 dB) condition tended to be greater than the 80-ms (−0.88 ± 1.4 dB; *p* = 0.053), and 280-ms delay conditions (−0.86 ± 1.5 dB; *p* < 0.05; Fisher’s least significant difference test). Moreover, the desynchronization was positively correlated with *Ownership* rating (*r* = 0.29, *p* < 0.01) and proprioceptive drift (*r* = 0.27, *p* < 0.05) across the conditions.

## Discussion

In the present study, we examined the effects of delayed visual feedback on illusory ownership of a fake (model) hand and EEG mu- and alpha-rhythm desynchronization elicited by fake hand movement. Both subjective ratings and proprioceptive drift evaluation demonstrated that participants felt illusory ownership toward the fake hand (i.e., RHI) in the 80-ms delay condition, but illusion strength significantly decreased as a function of the delay interval (i.e., in the 280 and 480-ms delay conditions). As shown in our previous study (Shibuya et al., 2018), we also found that participants sometimes produced spontaneous and unconscious finger movements when observing fake hand movement, and these movements occurred more frequently in the 80-ms than in the 480-ms delay condition.

Our previous study (Shibuya et al., 2018) compared EEG activity between the synchronous (RHI) and asynchronous conditions (non-RHI; visual feedback delay of 1 s) using channel-level analysis, and found that mu-rhythm desynchronization at the central electrode site (C3 or C4) during the fake hand movement was greater in the synchronous than asynchronous condition. On the other hand, to dissociate mu from occipital alpha, the current study applied ICA clustering to EEG data. Consequently, we obtained six clusters according to the predetermined criteria; bilateral sensorimotor clusters, bilateral occipital clusters, parietal cluster, and posterior cingulate cluster. Power decreases at ∼10 Hz were observed in all the clusters. Estimated dipole locations of the sensorimotor clusters, parietal cluster and occipital clusters corresponded to the premotor cortices (BA6), precuneus (medial region of right BA7) and visual cortices (BA18, 19), respectively, and these results were basically consistent with recent EEG and magnetoencephalography (MEG) studies incorporating ICA (Denis et al., 2017; Takahashi and Kitazawa, 2017). While neural oscillation of the sensorimotor and occipital regions is well-known as the mu- and alpha-rhythm, Takahashi and Kitazawa (2017) recently reported that strong source of the alpha-rhythm was located in the precuneus.

Of these clusters, a right sensorimotor cluster showed greater and more persistent mu-rhythm desynchronization in the 80-ms delay condition than in the 280 and 480-ms delay conditions, which was similar to what we observed in our previous study using channel data analysis (Shibuya et al., 2018). However, there were no differences among delay conditions in the bilateral occipital clusters. The difference between mu and occipital alpha components is an important finding, because some researchers have argued that mu-rhythm desynchronization during action observation is easily confounded with occipital alpha-rhythm desynchronization (Hobson and Bishop, 2016, 2017), which is modulated by attentional engagement (Perry and Bentin, 2010). Our findings refute the possibility that delay-dependent mu-rhythm desynchronization resulted from differences in attentional engagement to the fake hand movement, and again support that observing the embodied fake hand movements activates sensorimotor system of the observer.

We obtained other three clusters relating to the action observation. Of them, there was a left sensorimotor cluster ipsilateral to the stroked hand. This result is not surprising because a bilateral mu-suppression occurs over ipsilateral and contralateral central regions during the actual performance of the movement (Pineda, 2005). However, there were no significant differences in mu-rhythm desynchronization in the left sensorimotor cluster among the delay conditions. Indeed, it is known that mu-rhythm in each hemisphere is not coherent with each other (Pineda, 2005). Similarly, there was no significant delay-dependent modulation in the posterior cingulate cluster and the parietal cluster.

Although mu-rhythm desynchronization of the right sensorimotor cluster was significantly reduced from the 80 to the 280-ms delay condition, the delay-dependent modulation was slightly different from a sense of ownership toward the illusory hand. In the sense of ownership, the decrease from 80- to 280-ms delay was more evident for proprioceptive drift (*p* < 0.01) than for ownership rating (*p* = 0.08). This difference may reflect a distinction between subjective ownership rating and proprioceptive drift (i.e., altered body representation), though a mild correlation was identified between the two variables (*r* = 0.33, *p* < 0.01). Indeed, some studies have suggested that subjective ratings and proprioceptive drift are distinct entities (Holle et al., 2011; Rohde et al., 2011; Abdulkarim and Ehrsson, 2016; Shibuya et al., 2017). In line with our result, Shimada et al. (2014) also suggested a possibility that proprioceptive drift is more sensitive to the visual feedback delay, compared to ownership rating. Nevertheless, both ownership rating and proprioceptive drift further declined from the 280-ms delay condition to the 480-ms delay condition, whereas the mu-rhythm desynchronization showed no difference between the 280 and 480-ms delay conditions, which show disappearance of strong and persistent mu-rhythm desynchronization at 280-ms delay interval. This finding might suggest a difference of tolerance for visual feedback delay on the emergence of the mu-rhythm desynchronization and body ownership.

In our previous study (Shibuya et al., 2018), we hypothesized that higher sensorimotor activation (i.e., mu-rhythm desynchronization) during the observation of illusory embodied fake hand movement occurred to resolve inter-sensory conflict between visual (“my fingers are spreading”) and proprioceptive hand information (“my fingers are not spreading”), to maintain one’s own body image. This conflict would be resolved by altering the actual hand posture (i.e., proprioceptive information) to be consistent with that of the fake hand (i.e., visual information) (Asai, 2015) according to visual dominance theory (van Beers et al., 1996, 1999). Based on our hypothesis, the current findings suggest a possibility that the time window to drive the conflict-resolution processing (i.e., multisensory integration) during action observation could be narrower (stricter) than the time window for the RHI *per se* (ca. 200–300 ms; Shimada et al., 2009, 2014), though both are modulated by visual feedback delays. Given that the dipole centroid location of the right sensorimotor cluster was localized to BA 6, the greater mu-rhythm desynchronization (conflict-resolution processing) may have been related to premotor cortex (PM) activity. Indeed, the PM is an ideal candidate for multisensory integration of visual, tactile, and proprioceptive information regarding hand position and orientation in space (Graziano et al., 1997; Rizzolatti et al., 1997; Graziano, 1999; Lloyd et al., 2003). Moreover, previous studies using functional magnetic resonance imaging (fMRI) have showed a close relationship between the PM activity and body ownership (Ehrsson et al., 2004, 2005; Brozzoli et al., 2012; Gentile et al., 2015). For example, using the RHI paradigm, Ehrsson et al. (2004) found that the PM activity reflected the feeling of ownership of the fake hand, suggesting that multisensory integration in the PM cortex play a crucial role in hand ownership. Given the difference between mu-rhythm desynchronization and illusory ownership of the fake hand at 280-ms delay interval, our results were slightly different from these previous fMRI studies. However, a recent study by using repetitive transcranial magnetic stimulation reveals that suppression of the ventral PM affect the explicit detection of the visuo-tactile congruence without interfering with the RHI (Peviani et al., 2018).

We found that spontaneous finger movements during action observation occurred more frequently in the 80-ms delay (RHI) condition than in the 480-ms delay (non-RHI) condition. This result is basically consistent with our previous study (Shibuya et al., 2018) in which finger movements were induced more frequently in the synchronous condition (RHI) than the asynchronous condition (non-RHI). Additionally, the incidence of finger movement decreased as a function of delay interval length and consequently correlated with ownership rating (*r* = 0.43, *p* < 0.01) and proprioceptive drift (*r* = 0.24, *p* < 0.05). These results provide indirect evidence that illusory ownership of the fake hand was relevant to sensorimotor activation during the observation of fake hand movement. Although PM activity and its projections to the primary motor cortex are potentially implicated, PM activity could not explain the movements and illusory ownership by itself, as described above. The clusters obtained in the current study might contribute together with the PM. The precuneus, for example, is known as a part of a default mode network and shows negative blood-oxygen-level-dependent (BOLD) responses during movement (Nakata et al., 2019). Further investigations are necessary to inform the exact neural mechanism(s) underlying spontaneous finger movements.

Similar to the incidence of finger movements, subjective ratings of finger movements (*Awareness*) in the 80-ms delay condition were significantly higher than the 480-ms delay condition, in spite of high inter-subject variability. Additionally, the *Awareness* rating was positively correlated with the incidence of finger movements (*r* = 0.37, *p* < 0.01), showing a moderate association between awareness of the movements and the incidence. Some participants showed affirmative responses on the *Awareness* rating (≥+1) (e.g., 39%; 13/33 in 80-ms condition). Therefore, we infer that the participants attributed agency for the involuntary movements to the model (hand), because the *Awareness* rating was highly correlated with the *Agency control* rating (e.g., “I felt as if the model hand was controlling my hand movements”) (*r* = 0.83, *p* < 0.01), which significantly decreased from 80 to 480-ms delay condition again.

## Data Availability Statement

The datasets generated for this study are available on request to the corresponding author.

## Ethics Statement

This study was approved by the Institutional Review Board of the School of Medicine, Kyorin University and conducted in accordance with the principles and guidelines of the Declaration of Helsinki. All participants provided written informed consent prior to experiments in accordance with institutional guidelines.

## Author Contributions

All authors conceived and designed the experiment and discussed the results. SShib and SU performed the experiment and analyzed the data. SShib and YO wrote the manuscript.

## Conflict of Interest

The authors declare that the research was conducted in the absence of any commercial or financial relationships that could be construed as a potential conflict of interest.

## Abbreviations

EEG: electroencephalography
ERSP: event-related spectrum perturbation
ICA: independent component analysis
RHI: rubber hand illusion.

## References

[B1] Abdulkarim Z. Ehrsson H. H. (2016). No causal link between changes in hand position sense and feeling of limb ownership in the rubber hand illusion. *Atten. Percept. Psychophys.* 78 707–720. 10.3758/s13414-015-1016-0 26555651 PMC4744264

[B2] Aizu N. Oouchida Y. Izumi S. (2018). Time-dependent decline of body-specific attention to the paretic limb in chronic stroke patients. *Neurology* 91 e751–e758. 10.1212/WNL.0000000000006030 30054442

[B3] Artoni F. Menicucci D. Delorme A. Makeig S. Micera S. (2014). RELICA: a method for estimating the reliability of independent components. *Neuroimage* 103 391–400. 10.1016/j.neuroimage.2014.09.010 25234117 PMC6656895

[B4] Asai T. (2015). Illusory body-ownership entails automatic compensative movement: for the unified representation between body and action. *Exp. Brain Res.* 233 777–785. 10.1007/s00221-014-4153-0 25424866

[B5] Bell A. J. Sejnowski T. J. (1995). An information-maximization approach to blind separation and blind deconvolution. *Neural Comput.* 7 1129–1159. 10.1162/neco.1995.7.6.1129 7584893

[B6] Botvinick M. Cohen J. (1998). Rubber hands ‘feel’ touch that eyes see. *Nature* 391:756. 10.1038/35784 9486643

[B7] Brozzoli C. Gentile G. Ehrsson H. H. (2012). That’s near my hand! parietal and premotor coding of hand-centered space contributes to localization and self-attribution of the hand. *J. Neurosci.* 32 14573–14582. 10.1523/JNEUROSCI.2660-12.2012 23077043 PMC6621451

[B8] Burin D. Garbarini F. Bruno V. Fossataro C. Destefanis C. Berti A. (2017). Movements and body ownership: evidence from the rubber hand illusion after mechanical limb immobilization. *Neuropsychologia* 107 41–47. 10.1016/j.neuropsychologia.2017.11.004 29109038

[B9] Burin D. Livelli A. Garbarini F. Fossataro C. Folegatti A. Gindri P. (2015). Are movements necessary for the sense of body ownership? evidence from the rubber hand illusion in pure hemiplegic patients. *PLoS One* 10:e0117155. 10.1371/journal.pone.0117155 25775041 PMC4361688

[B10] Delorme A. Makeig S. (2004). EEGLAB: an open source toolbox for analysis of single-trial EEG dynamics including independent component analysis. *J. Neurosci. Methods* 134 9–21. 10.1016/j.jneumeth.2003.10.009 15102499

[B11] Denis D. Rowe R. Williams A. M. Milne E. (2017). The role of cortical sensorimotor oscillations in action anticipation. *Neuroimage* 146 1102–1114. 10.1016/j.neuroimage.2016.10.022 27746385

[B12] Ehrsson H. H. Holmes N. P. Passingham R. E. (2005). Touching a rubber hand: feeling of body ownership is associated with activity in multisensory brain areas. *J. Neurosci.* 25 10564–10573. 10.1523/JNEUROSCI.0800-05.2005 16280594 PMC1395356

[B13] Ehrsson H. H. Spence C. Passingham R. E. (2004). That’s my hand! activity in premotor cortex reflects feeling of ownership of a limb. *Science* 305 875–877. 10.1126/science.1097011 15232072

[B14] Gallagher S. (2000). Philosophical conceptions of the self: implications for cognitive science. *Trends Cogn. Sci.* 4 14–21. 10.1016/S1364-6613(99)01417-5 10637618

[B15] Gallagher S. (2005). *How the Body Shapes the Mind.* New York, NY: Oxford University Press, 10.1093/0199271941.001.0001

[B16] Gentile G. Bjornsdotter M. Petkova V. I. Abdulkarim Z. Ehrsson H. H. (2015). Patterns of neural activity in the human ventral premotor cortex reflect a whole-body multisensory percept. *Neuroimage* 109 328–340. 10.1016/j.neuroimage.2015.01.008 25583608 PMC4349631

[B17] Graziano M. S. (1999). Where is my arm? the relative role of vision and proprioception in the neuronal representation of limb position. *Proc. Natl. Acad. Sci. U.S.A.* 96 10418–10421. 10.1073/pnas.96.18.10418 10468623 PMC17903

[B18] Graziano M. S. Hu X. T. Gross C. G. (1997). Coding the locations of objects in the dark. *Science* 277 239–241. 10.1126/science.277.5323.239 9211852

[B19] Hobson H. M. Bishop D. V. (2016). Mu suppression - a good measure of the human mirror neuron system? *Cortex* 82 290–310. 10.1016/j.cortex.2016.03.019 27180217 PMC4981432

[B20] Hobson H. M. Bishop D. V. (2017). The interpretation of mu suppression as an index of mirror neuron activity: past, present and future. *R. Soc. Open Sci.* 4:160662. 10.1098/rsos.160662 28405354 PMC5383811

[B21] Holle H. McLatchie N. Maurer S. Ward J. (2011). Proprioceptive drift without illusions of ownership for rotated hands in the “rubber hand illusion” paradigm. *Cogn. Neurosci.* 2 171–178. 10.1080/17588928.2011.603828 24168532

[B22] Kalckert A. Ehrsson H. H. (2012). Moving a rubber hand that feels like your own: a dissociation of ownership and agency. *Front. Hum. Neurosci.* 6:40. 10.3389/fnhum.2012.00040 22435056 PMC3303087

[B23] Lancaster J. L. Rainey L. H. Summerlin J. L. Freitas C. S. Fox P. T. Evans A. C. (1997). Automated labeling of the human brain: a preliminary report on the development and evaluation of a forward-transform method. *Hum. Brain Mapp.* 5 238–242. 20408222 10.1002/(SICI)1097-0193(1997)5:4<238::AID-HBM6>3.0.CO;2-4 PMC2860189

[B24] Lancaster J. L. Woldorff M. G. Parsons L. M. Liotti M. Freitas C. S. Rainey L. (2000). Automated talairach atlas labels for functional brain mapping. *Hum. Brain Mapp.* 10 120–131. 10.1002/1097-0193(200007)10:3<120::AID-HBM30>3.0.CO;2-8 10912591 PMC6871915

[B25] Lloyd D. M. Shore D. I. Spence C. Calvert G. A. (2003). Multisensory representation of limb position in human premotor cortex. *Nat. Neurosci.* 6 17–18. 10.1038/nn991 12483217

[B26] MacQueen J. (1967). “Some methods for classification and analysis of multivariate observations,” in *Proceedings of the 5th Berkeley Symposium on Mathematical Statistics and Probability*, eds Le Cam L. M. Neyman J., (Berkeley, CL: University of California Press), 281–297.

[B27] Makeig S. Westerfield M. Jung T. P. Enghoff S. Townsend J. Courchesne E. (2002). Dynamic brain sources of visual evoked responses. *Science* 295 690–694. 10.1126/science.1066168 11809976

[B28] Nakata H. Domoto R. Mizuguchi N. Sakamoto K. Kanosue K. (2019). Negative BOLD responses during hand and foot movements: an fMRI study. *PLoS One* 14:e0215736. 10.1371/journal.pone.0215736 31002697 PMC6474656

[B29] Oberman L. M. Hubbard E. M. McCleery J. P. Altschuler E. L. Ramachandran V. S. Pineda J. A. (2005). EEG evidence for mirror neuron dysfunction in autism spectrum disorders. *Brain Res. Cogn. Brain Res.* 24 190–198. 10.1016/j.cogbrainres.2005.01.014 15993757

[B30] Oldfield R. C. (1971). The assessment and analysis of handedness: the edinburgh inventory. *Neuropsychologia* 9 97–113. 10.1016/0028-3932(71)90067-4 5146491

[B31] Perry A. Bentin S. (2010). Does focusing on hand-grasping intentions modulate electroencephalogram mu and alpha suppressions? *Neuroreport* 21 1050–1054. 10.1097/WNR.0b013e32833fcb71 20838261

[B32] Peviani V. Magnani F. G. Ciricugno A. Vecchi T. Bottini G. (2018). Rubber hand illusion survives ventral premotor area inhibition: a rTMS study. *Neuropsychologia* 120 18–24. 10.1016/j.neuropsychologia.2018.09.017 30266289

[B33] Pineda J. A. (2005). The functional significance of mu rhythms: translating “seeing” and “hearing” into “doing”. *Brain Res. Brain Res. Rev.* 50 57–68. 10.1016/j.brainresrev.2005.04.005 15925412

[B34] Rizzolatti G. Fadiga L. Fogassi L. Gallese V. (1997). The space around us. *Science* 277 190–191. 10.1126/science.277.5323.190 9235632

[B35] Rohde M. Di Luca M. Ernst M. O. (2011). The rubber hand illusion: feeling of ownership and proprioceptive drift do not go hand in hand. *PLoS One* 6:e21659. 10.1371/journal.pone.0021659 21738756 PMC3125296

[B36] Schutz-Bosbach S. Mancini B. Aglioti S. M. Haggard P. (2006). Self and other in the human motor system. *Curr. Biol.* 16 1830–1834. 10.1016/j.cub.2006.07.048 16979561

[B37] Shibuya S. Unenaka S. Ohki Y. (2017). Body ownership and agency: task-dependent effects of the virtual hand illusion on proprioceptive drift. *Exp. Brain Res.* 235 121–134. 10.1007/s00221-016-4777-3 27651139

[B38] Shibuya S. Unenaka S. Zama T. Shimada S. Ohki Y. (2018). Spontaneous imitative movements induced by an illusory embodied fake hand. *Neuropsychologia* 111 77–84. 10.1016/j.neuropsychologia.2018.01.023 29407592

[B39] Shimada S. Fukuda K. Hiraki K. (2009). Rubber hand illusion under delayed visual feedback. *PLoS One* 4:e6185. 10.1371/journal.pone.0006185 19587780 PMC2702687

[B40] Shimada S. Qi Y. Hiraki K. (2010). Detection of visual feedback delay in active and passive self-body movements. *Exp. Brain Res.* 201 359–364. 10.1007/s00221-009-2028-6 19830411

[B41] Shimada S. Suzuki T. Yoda N. Hayashi T. (2014). Relationship between sensitivity to visuotactile temporal discrepancy and the rubber hand illusion. *Neurosci. Res.* 85 33–38. 10.1016/j.neures.2014.04.009 24874005

[B42] Slater M. Perez-Marcos D. Ehrsson H. H. Sanchez-Vives M. V. (2008). Towards a digital body: the virtual arm illusion. *Front. Hum. Neurosci.* 2:6. 10.3389/neuro.09.006.2008 18958207 PMC2572198

[B43] Takahashi T. Kitazawa S. (2017). Modulation of illusory reversal in tactile temporal order by the phase of posterior alpha rhythm. *J. Neurosci.* 37 5298–5308. 10.1523/JNEUROSCI.2899-15.2017 28450538 PMC6596459

[B44] Tsakiris M. Hesse M. D. Boy C. Haggard P. Fink G. R. (2007). Neural signatures of body ownership: a sensory network for bodily self-consciousness. *Cereb. Cortex* 17 2235–2244. 10.1093/cercor/bhl131 17138596

[B45] van Beers R. J. Sittig A. C. Gon J. J. (1999). Integration of proprioceptive and visual position-information: an experimentally supported model. *J. Neurophysiol.* 81 1355–1364. 10.1152/jn.1999.81.3.1355 10085361

[B46] van Beers R. J. Sittig A. C. van der Gon J. J. (1996). How humans combine simultaneous proprioceptive and visual position information. *Exp. Brain Res.* 111 253–261. 10.1007/bf00227302 8891655

